# Spectrum of Microbial Diseases and Resistance Patterns at a Private Teaching Hospital in Kenya: Implications for Clinical Practice

**DOI:** 10.1371/journal.pone.0147659

**Published:** 2016-01-25

**Authors:** Daniel Maina, Geoffrey Omuse, Gunturu Revathi, Rodney D. Adam

**Affiliations:** 1 Department of Pathology, Aga Khan University Hospital, Nairobi, Kenya; 2 Department of Medicine, Aga Khan University Hospital, Nairobi, Kenya; Leibniz-Institute DSMZ, GERMANY

## Abstract

**Background:**

Accurate local prevalence of microbial diseases and microbial resistance data are vital for optimal treatment of patients. However, there are few reports of these data from developing countries, especially from sub-Saharan Africa. The status of Aga Khan University Hospital Nairobi as an internationally accredited hospital and a laboratory with an electronic medical record system has made it possible to analyze local prevalence and antimicrobial susceptibility data and compare it with other published data.

**Methods:**

We have analyzed the spectrum of microbial agents and resistance patterns seen at a 300 bed tertiary private teaching hospital in Kenya using microbial identity and susceptibility data captured in hospital and laboratory electronic records between 2010 and 2014.

**Results:**

For blood isolates, we used culture collection within the first three days of hospitalization as a surrogate for community onset, and within that group, *Escherichia coli* was the most common, followed by *Staphylococcus aureus*. In contrast, *Candida spp*. and *Klebsiella pneumoniae* were the most common hospital onset causes of bloodstream infection. Antimicrobial resistance rates for the most commonly isolated Gram negative organisms was higher than many recent reports from Europe and North America. In contrast, Gram positive resistance rates were quite low, with 94% of *S*. *aureus* being susceptible to oxacillin and only rare isolates of vancomycin-resistant enterococci.

**Conclusions:**

The current report demonstrates high rates of antimicrobial resistance in Gram negative organisms, even in outpatients with urinary tract infections. On the other hand, rates of resistance in Gram positive organisms, notably *S*. *aureus*, are remarkably low. A better understanding of the reasons for these trends may contribute to ongoing efforts to combat antimicrobial resistance globally.

## Introduction

Treatment of suspected bacterial infections is frequently initiated on the basis of clinical presentation, especially in acutely ill patients. Laboratory tests are performed and therapy is subsequently modified based on microbial results and the clinical course. However, accurate initial empiric therapy depends on understanding of local disease prevalence and susceptibility patterns. Disease prevalence information can identify both similarities and differences in microbial etiologies of clinical syndromes when compared with other regions. The modifications to the initial empiric therapy depend heavily on timely and accurate microbial data.

When laboratory diagnoses are not available, clinical decision making is based on knowledge or assumptions regarding prevalent pathogens. In sub-Saharan Africa, it is common to treat acute febrile illness as malaria, even in areas such as Nairobi where there is little or no malaria transmission. When these assumptions are incorrect, morbidity and mortality result from missed diagnoses. For example, a study of 251 children with a presumed clinical diagnosis of severe malaria in Ghana found a high rate of bacteremia (20%) [1]. The mortality in the bacteremic group was 39% versus 9.7% for blood culture negative children. This study points out the compromises to clinical care that can result from lack of accurate and timely laboratory diagnosis of infectious illnesses. It also demonstrates the potential value of having accurate and up to-date local information on the spectrum of infectious illnesses. While hospital laboratories are unable to provide the depth of information that results from community-wide prospective surveillance, they can point to key trends [2].

There is relatively little published information on levels of antimicrobial resistance within sub-Saharan Africa. Trends within the region could have varying types of impact on resistance levels. For example, the availability of broad spectrum antibiotics is limited by cost in a vast portion of the public sector which could decrease resistance. However, antimicrobial agents are frequently available without prescriptions and are also used commonly in agriculture for food animals. In addition, trimethoprim/sulfamethoxazole is widely used for *Pneumocystis* prophylaxis in HIV-infected patients. These practices all have the potential to increase resistance levels.

In this report, we have studied the microbiology and resistance patterns of infectious diseases seen at AKUHN, illuminating both similarities and differences compared to North American and European data.

## Materials and Methods

AKUHN is a 300 bed university hospital in Nairobi Kenya with a range of post-graduate medical education programs. The patients are primarily from the upper middle and high socioeconomic groups comprised mainly of African Kenyans, Kenyans of Asian descent and a small Caucasian population- mostly expatriates. The moderate numbers of HIV-infected patients seen at the hospital reflect the national HIV prevalence of nearly 7%, which is midrange for sub-Saharan Africa. The hospital has about 50 critical beds, including intensive care unit, coronary care unit, cardiothoracic intensive care unit, neonatal intensive care unit, and high dependency units. There is a nine bed outpatient dialysis unit and a cancer center. The hospital conducts approximately 3600 deliveries in a year.

### Microbiology and antigen testing

The hospital laboratory is ISO 15189 accredited by the South Africa national accreditation service (SANAS)- the first hospital laboratory in East Africa to achieve this [3]. The microbiology laboratory performs a range of aerobic bacteriology, mycology and mycobacteriology tests. The laboratory uses an automated detection system (BD BACTEC™) for blood cultures, and VITEK® 2 (bioMérieux, France) for microbial identification and susceptibility testing. The 2009 Clinical Laboratory Standards Institute (CLSI) M100-S19 breakpoints were used for interpretation. When carbapenem resistance was identified by Vitek testing, the results were routinely confirmed by disk diffusion using an imipenem disk. Supplementary manual methods are also available, including serotyping to identify Salmonella serovars and streptococcal species. In cases where automated susceptibilities are not defined, disk diffusion (Oxoid™ disks) or E-test® (bioMérieux, France) methods are used. Entero-pathogenic *Escherichia coli* (EPEC) testing was performed using commercial antisera obtained from Remel Laboratories (Lenexa, Kansas, USA; part of Thermo Scientific). *Clostridium difficile* antigen detection was done using the Meridian Immunocard (Meridian Bioscience; Inc. USA), which detects toxins A and B. A series of studies of this test revealed sensitivities ranging from 75% to 98%, while specificities ranged from 96 to 100% [4].

### Data Analysis

Laboratory data have been available since September 2010 on the hospital/laboratory information system. The database includes patient location, sample type, and date of hospital admission (for hospitalized patients). We used this database for microbial prevalence data, excluding organisms of given sample types that were repeated within a one month period. Antimicrobial susceptibility data were more complete when obtained directly from the Vitek2 database, and were available continuously from 2012 through 2014. Within each year, we included only the first isolation of an organism from a specific sample type. Data are reported by the percent that are completely susceptible as modified by the Vitek2 algorithm for inferring resistance to specific antimicrobial agents on the basis of resistance to other drugs in the same class.

All positive cultures from September 2010 through 2014 were extracted from the hospital information system. Positive cultures were accompanied by relevant demographic data including age and sex, inpatient or outpatient and when inpatient, the hospital location of the culture request, dates of cultures request and hospital admission (for inpatients). When there were multiple positive cultures from a single source in a single episode of infection, only the first positive result was included in the analysis. When two cultures with the same organism were collected more than 30 days apart in the absence of an intervening positive culture, it was considered as a second episode. Unless stated otherwise, only microbiology samples from the hospital campus were included to avoid referral biases for other sources of samples.

We analyzed the data with the objective of determining which pathogenic organisms were associated with community onset or with hospital-acquired infection using the collection of the culture within the first three days of hospitalization versus later as a surrogate marker for community onset vs. hospital onset. Hospital onset infections are typically defined as those with symptom onset after at least 48 hours in the hospital. Accurate categorization requires extensive evaluation of individual patients [5], but for BSI, a culture drawn either two or three days after admission is highly predictive of hospital acquired infection [6]. We chose a three day cutoff to allow for a potential one day delay in obtaining the blood culture.

### Ethics Review

The study was reviewed and approved by the clinical research ethics committee of Aga Khan University Nairobi, Kenya. All individual patient-identifying information was removed prior to analysis and as such, the study was granted a waiver of requirement for individual consent by the ethics review committee.

## Results

### Etiologies of Blood Stream Infections (BSI)

The most common isolate from either group was coagulase negative staphylococci (ConS). ConS are nonpathogens the majority of time, but can also be pathogenic. When pathogenic, it is usually in the setting of an intravascular device and supported by at least two positive cultures. Since the clinical information was not available for this analysis, and because most of the time, just a single blood culture had been obtained, these isolates were excluded from further analysis. Of note, 77% of the ConS obtained from blood cultures in 2014 were resistant to oxacillin. Likewise, viridans streptococcal blood isolates are nonpathogenic the majority of the time and were excluded from analysis.

### Etiologies of community onset bloodstream infection

Of the organisms causing bacteremia within 72 hours of hospital admission, *E*. *coli* was the most common, followed by *S*. *aureus* and *S*. *pneumoniae* (together with *K*. *pneumoniae*) respectively (Table 1). It is only when surveying the less common blood isolates that the spectrum is significantly different from what is found in developed countries. Notably, *Salmonella species* (both typhoidal and non-typhoidal) comprised nearly 6% of community-acquired isolates. We also considered whether the age of our patients was an important contributing factor in view of the fact that many of the reported series were primarily from children, and the patient population in the current series was older. Therefore, we compared the ages of patients with bacteremia caused by *E*. *coli*, *Klebsiella* and *Salmonella*. The average ages were 57, 40 and 30 years, respectively. When limiting the analysis to patients whose blood cultures were ordered within 3 days of hospitalization, the ages were 59, 46, and 28 years, respectively. Because of the large number of neonatal bacteremia cases caused by Klebsiella, we also determined the average ages when excluding patients less than one year of age and found that the mean ages for *E*. *coli*, *Klebsiella* and *Salmonella* bacteremia were 60, 56 and 32, respectively, demonstrating that the age at time of bacteremia for *Klebsiella* was disproportionately affected by the neonatal infections. Similarly, *Cryptococcus neoformans* comprised 3% of community onset BSI, all or nearly all associated with HIV infection.

**Table 1:** Etiologies of bloodstream infections.

Community Onset			Hospital Onset		
Organism	#	%	Organism	#	%
*Escherichia coli*	124	31%	*Candida spp*.	143	34%
*Staphylococcus aureus*	65	16%	*Klebsiella pneumoniae*	74	17%
*Klebsiella pneumoniae*	34	8%	*Escherichia coli*	52	12%
*Streptococcus pneumoniae*	34	8%	*Enterococcus spp*.	45	11%
*Enterococcus spp*.	19	5%	*Staphylococcus aureus*	27	6%
*Pseudomonas aeruginosa*	19	5%	*Acinetobacter baumannii*	21	5%
*Streptococcus agalactiae* (Gp B strep)	15	4%	*Enterobacter spp*.	20	5%
*Candida spp*.	14	3%	*Pseudomonas aeruginosa*	16	4%
*Cryptococcus neoformans*	12	3%	Salmonella Typhimurium	6	1%
Salmonella Typhimurium	12	3%			
Salmonella Typhi	11	3%			

Other community-onset organisms with between 5 and 10 episodes include *Streptococcus pyogenes* (9), *Streptococcus dysgalactiae* (6), *A*. *baumannii* (5), *Enterobacter spp*. (5) and Group D streptococci (5).

### Etiologies of hospital acquired bloodstream infection

*Candida* species were the most common etiologies of hospital onset BSI, with 73% consisting of non-albicans *Candida spp*. It is also worth noting that *S*. *aureus* was only the fifth most common cause of hospital onset BSI and that *S*. *aureus* was much more likely to be a community-onset rather than hospital-onset pathogen.

### Etiologies of Neonatal BSI

We evaluated neonatal BSI differently than the “all BSI” group, taking into account the understanding of the variable definitions of neonatal sepsis. Traditionally, sepsis occurring in the first month of life has been divided into early (= < 7 days) and late sepsis (8 days to one month). Alternatively, particularly for premature neonates, it may be divided into sepsis occurring within the first three days or sepsis occurring later than that [7]. Thus, we have divided our results into sepsis occurring within the first three days, in 4–7 days, or greater than 7 days (Table 2). *S*. *agalactiae* sepsis occurred exclusively within the first 3 days of life while the majority of *E*. *coli*, *K*. *pneumoniae* and enterococcal cases occurred later than three days. The majority of cases occurring after seven days were premature infants in the newborn intensive care unit, but some were of community onset in neonates who were admitted to the hospital after seven days of age. Three *S*. *aureus*, two *K*. *pneumoniae*, one *S*. *pyogenes*, and one *S*. *pneumoniae* episode fell into this class. Enterococci were common in the early and late periods, but how many of these were clinically significant is not known since they are also common as contaminants. In contrast to adults, Candida species were not a major cause of BSI.

**Table 2:** Etiologies of neonatal bloodstream infection.

Organism	< = 3d	4-7d	>7d	Total
*Klebsiella pneumoniae*	1	8	12	21
*Enterococcus spp*.	3	2	9	14
*Streptococcus agalactiae* (Gp B strep)	9	0	0	9
*Escherichia coli*	1	4	2	7
*Enterobacter spp*.	1	2	3	6
*Staphylococcus aureus*	1	0	4	5
Streptococcus Group D	3	0	0	3
*Pseudomonas aeruginosa*	0	1	2	3
*Streptococcus pneumoniae*	1	0	1	2
*Candida spp*.	0	0	2	2
*Corynebacterium jeikeium*	1	0	0	1
*Listeria monocytogenes*	1	0	0	1
*Acinetobacter baumannii*	0	0	1	1
*Burkholderia cepacia*	0	1	0	1
*Serratia marcescens*	0	0	1	1
*Streptococcus pyogenes*	0	0	1	1

### Urinary Tract Infections in outpatients

The spectrum of isolates was similar to that reported elsewhere in the world, with *E*. *coli* as the leading isolate and *Klebsiella spp*. a distant second (Table 3).

**Table 3:** Etiologies of urinary and gastrointestinal infections.

Urine isolates (2014 only)		Stool Isolates (Sept 2010 through 2014)		
ORGANISM	%	Organism	< = 5 yrs (n = 356)	>5 yrs (n = 455)
*Escherichia`coli*	76%	Salmonella Typhimurium	33%	54%
*Klebsiella spp*.	11%	*Shigella species*	22%	41%
*Staphylococcus saprophyticus*	2%	EPEC	42%	0%
*Enterococcus spp*.	2%	*Campylobacter species*	3%	3%
*Proteus spp*.	2%	Salmonella Paratyphi	1%	2%
*Morganella morganii*	2%	Salmonella Typhi	0%	1%
		*Vibrio cholerae*	0%	0%

### Stool pathogens

Testing for EPEC was performed only on children under five years of age, so the table is divided into the two age groups. In children under five, EPEC was the most common pathogen at 41%, but *Salmonella spp*. and *Shigella spp*. were also found commonly. In patients over five, *Salmonella spp*. and *Shigella spp*. were the predominant bacterial pathogens identified in stool with over 30% for each (Table 3). Salmonella Typhi and Salmonella Paratyphi were rarely identified from stool cultures, but comprised about half the BSIs due to Salmonella species (Table 1). The stool samples represented a combination of outpatient and hospitalized patients with 19% of positive samples obtained from hospitalized patients (27% of positive samples from children under five and 13% of positive samples for patients over five). The frequency and severity of *C*. *difficile* disease in this region has not been reported. Of 1397 *C*. *difficile* Toxin A and B antigen tests done between 2008 and 2015, 2.6% were positive. This prevalence rate is low enough that these could all be false positive results. A systematic review found that the specificity of the test used varied from 94 to 99% in 9 reports, with an average of 97% [4].

### Bacterial Resistance

#### Gram negative organisms

The best window into the community for resistance levels in certain bacteria is urinary tract cultures from outpatients. Thus, we analyzed the susceptibilities of urinary pathogens found in specimens from outpatients seen from the years 2012 through 2014 (Table 4). The data are shown separately for each year and overall, there was little change from year to year. For patients with more than one urine culture demonstrating the same isolate, we included only the first within a calendar year. The rate of resistance to a broad range of antibiotics is striking. The susceptibility in 2014 of only 23% of *E*. *coli* and 45% of *Klebsiella spp*. to TMP/SMX and the susceptibility of only 22% of *E*. *coli* isolates to ampicillin may reflect the widespread use of these drugs throughout the country. TMP/SMX is routinely recommended for *Pneumocystis* prophylaxis in HIV-infected patients and ampicillin/amoxicillin is commonly used for respiratory illness. However, susceptibility rates are also low for other classes of drugs as well, including 79% and 65% to third generation cephalosporins for *E*. *coli* and *Klebsiella*, respectively, and 66 and 76% for quinolones. For lower urinary tract infections caused by *E*. *coli*, the best susceptibility among orally administered drugs was nitrofurantoin (86%).

**Table 4:** Susceptibilities of Gram negative organisms: 2012 to 2014.

Organism	Source	Year	Number	Amox	Amox/Clav	TMP/SMX	Cipro	Cefurox	Cefotax	Gent	Amikacin	Mero	Ntfn
*E*. *coli*	Urine	2012	1047	20%	66%	24%	68%	78%	83%	84%	100%	100%	86%
*E*. *coli*	Urine	2013	903	21%	68%	25%	66%	76%	81%	84%	100%	100%	86%
*E*. *coli*	Urine	2014	962	22%	68%	23%	66%	74%	79%	85%	100%	100%	86%
*K*. *pneumoniae*	Urine	2012	135		56%	39%	84%	69%	72%	79%	99%	99%	
*K*. *pneumoniae*	Urine	2013	125		52%	45%	77%	58%	62%	69%	99%	98%	
*K*. *pneumoniae*	Urine	2014	105		53%	45%	76%	62%	65%	74%	100%	99%	
*P*. *mirabilis*	Urine	All	81	35%	83%	33%	93%	95%	99%	89%	100%	99%	
*E*. *coli*	Blood	2012	48	10%	50%	15%	33%	42%	46%	79%	98%	100%	
*E*. *coli*	Blood	2013	44	2%	39%	11%	48%	46%	52%	64%	100%	100%	
*E*. *coli*	Blood	2014	47	15%	43%	19%	43%	53%	55%	64%	100%	100%	
*K*. *pneumoniae*	Blood	2012	31		36%	23%	61%	29%	36%	42%	100%	97%	
*K*. *pneumoniae*	Blood	2013	25		32%	32%	64%	32%	36%	48%	88%	92%	
*K*. *pneumoniae*	Blood	2014	27		26%	11%	52%	11%	15%	22%	85%	70%	

Gram negative blood isolates were a mixture of community and hospital onset infections. Thus, the lower susceptibility rates likely reflect the greater number of hospital-acquired isolates. Seventy percent of the *E*. *coli* blood isolates were community onset, but even so, the susceptibility rates in 2014 for cephalosporins and quinolones were substantially lower for blood than for urine isolates; 55% vs. 74% for third generation cephalosporins and 43% vs. 66% for quinolones. The difference was more pronounced for *Klebsiella spp*. for which 69% were hospital onset. For 2014 *Klebsiella spp*. isolates, 65% of urine, but only 15% of blood isolates were susceptible to third generation cephalosporins.

#### Gram positive organisms

In contrast to the Gram negative organisms, some of the key Gram positive organisms have much higher susceptibility rates than those reported from Europe or North America (Table 5). Ninety-four percent of *S*. *aureus* isolates were susceptible to oxacillin, while 84% and 87% were susceptible to tetracycline and erythromycin, respectively. All *E*. *faecalis* isolates were susceptible to ampicillin, while 57% and 71% demonstrated susceptibility to gentamicin and streptomycin for synergy purposes. Vancomycin-resistant *E*. *faecium* (VRE) was rarely seen, although the majority of *E*. *faecium* isolates were resistant to ampicillin (data not shown). During the period from 2012 through 2014, there were no significant changes in susceptibilities for these organisms.

**Table 5:** Susceptibilities of Gram positive organisms: 2012 to 2014.

Organism	Source	Year	Number	Amox (BLN)	Pen HS	Pen HS or IS	Ntfn	Ox	Tet	Eryt	Vanco	Linez	Gent syn	Strep syn
S. saprophyticus	Urine	2014	61	49%			100%							
*E*. *faecalis*	Urine	2014	45	98%							100%	98%		
*S*. *aureus*	Blood	2014	18	6%				94%	83%	94%	100%	100%		
*S*. *aureus*	All	2014	271	8%				94%	84%	87%	100%	99%		
*E*. *faecalis*	Blood	2012–2014	28	100%							100%	96%	57%	71%
*S*. *pneumoniae*	Blood	2012–2014	28		46%	100%			82%	93%	100%			
*S*. *pneumoniae*	All	2014	25		24%	84%			68%	64%	100%			
CoNS	Blood	2014	114	12%				20%		32%	100%	91%		

## Discussion

The results presented in this analysis of microbiological laboratory data provide the accuracy expected of an internationally accredited laboratory with a full range of internal quality as well as external quality assurance measures. The electronic database has made it possible to do a comprehensive analysis of the data for over four years (prevalence) and three years (susceptibilities).

For blood stream pathogens, we used a criterion of including cultures obtained within the first three days of hospitalization as a surrogate for community onset infections [6]. Since hospital-acquired infections are typically defined as those with symptom onset more than 48 hours after hospitalization, this approach allowed for a one day delay in obtaining the culture. This approach has the limitation of an occasional misclassification due to community onset infection with delayed blood culture, as demonstrated by the occasional Salmonella Typhimurium from blood cultures obtained more than three days into hospitalization. In addition, the finding of occasional cases of candidemia occurring within the first three days points to these infections as being medical care-related since candidemia is extremely rare in the absence of predisposing medical interventions. Although the proportion of patients with non-hospital medical exposure is less than in North America or Europe, there is still a number of patients with dialysis, chemotherapy, and other types of medical interventions. However, despite these limitations, the categories give an insight into the predominant organisms causing community versus hospital onset infections.

For community onset BSI, the top three organisms (Table 1) were similar to findings reported in several other countries. Three separate studies in Europe that analyzed characteristics of hospital-acquired and community-onset BSI in Austria, Spain and Portugal reported *E*. *coli*, *S*. *pneumoniae*, and *S*. *aureus* as the predominant organisms in community acquired BSI [8–10]. This was also reflected in a multicenter cohort study in community hospitals in the USA where a similar pattern was observed [11]. The major differences from North American or European series in the current study were in the less frequently seen micro-organisms, such as the *Salmonella species* and *C*. *neoformans*.

While the *Salmonella* percentage is higher than in European and American studies, it contrasts with the findings of a systematic review in developing countries of South and South East Asia, in which *Salmonella spp*. were the most isolated organisms in community acquired BSI [12]. A systematic review and meta-analysis of 22 studies conducted across Africa on community acquired blood stream infections reported *S*. *enterica* serotypes as the most and second most prevalent organisms in adults and children respectively. *S*. *pneumoniae* and *E*. *coli* were 2nd and 3rd in prevalence among adults, while *S*. *pneumoniae* was the most common organism isolated in children [13]. A portion of the difference of our results from these reports can be explained by the older population in the current series than in other reports as demonstrated. The age-related impact on proportion of *Salmonella spp*. is supported by the younger age in our population for *Salmonella spp*. bacteremia than for *E*. *coli* or *K*. *pneumoniae*. However, there is also a temporal trend at our institution as demonstrated in a previous publication from this institution [14] in which *Salmonella spp*. accounted for 15% of BSIs in HIV-infected patients and 13% in non-HIV-infected patients (14% of all BSI) from 2003 to 2008. The earlier series included both community onset and hospital onset BSI, so it contrasts significantly with the current series in which only about 3% of all BSIs were caused by *Salmonella spp*. There was also a temporal decrease in the portion of *Salmonella* BSIs due to Salmonella Typhi in that Salmonella Typhi caused 59% of *Salmonella* BSIs in the earlier series and only 38% in the current series.

Since cryptococcal fungemia is found almost exclusively in advanced HIV, its frequency dropped with the rollout of antiretroviral therapy in Europe and the US [15–17].

For hospital acquired BSIs, the major differences from other reports are the predominance of candidemia and the relatively infrequent isolation of *S*. *aureus*. In a surveillance of hospital acquired bacteremia in English hospitals, 26% of the isolates causing hospital-acquired bacteremia were *S*. *aureus* and 16% CoNS, followed by *E*. *coli* and *K*. *pneumoniae* [18]. A similar study in Finnish hospitals showed 65% of the isolates were gram positive led by CoNS, 31% Gram negative of which majority were *E*. *coli*, and 4% were fungal mainly *Candida spp*. [19].

The finding of *S*. *agalactiae* (Group B Strep) as the leading cause of early onset neonatal bacteremia is similar to the findings of most western series in which *S*. *agalactiae* is the leading cause of neonatal BSI [20, 21]. *E*. *coli* was much less common in the current series as a cause of early onset BSI, but accounted for a significant number of later onset BSI (five episodes; 11%). Three of the five occurred within the first seven days of hospitalization. Closer to home, a study that analyzed bacteremia among children admitted to a rural hospital in Kenya showed that among infants who were under 60 days old, *E*. *coli* and group B streptococci predominated among a broad range of isolates (14 percent and 11 percent, respectively) [22]. It is important to note that *K*. *pneumoniae* is a major pathogen in community and hospital onset BSIs and in adults as well as neonates. For neonates, it is the most common hospital onset pathogen and is also found occasionally as an early onset pathogen.

The susceptibility of *E*. *coli* and *Klebsiella spp*. obtained from blood cultures was much lower than reported in the West, with susceptibilities of 47% and 24% to third generation cephalosporins, respectively, and 41% and 59% for quinolones (Table 4). In contrast, data from hospitals throughout the United States showed that of the 483 *K*. *pneumoniae* isolates implicated in cases of device-associated BSI, 72.9% were susceptible to 3rd generation cephalosporins [23] and there was little change in a later survey {Sievert 2013}. A study of Gram negative organisms from ICUs in the US and Europe showed that about 90% of *E*. *coli* in the US and 86% in Europe were susceptible to third generation cephalosporins and that 85% and 73% of *Klebsiella*, respectively, were susceptible [24]. Among outpatient urinary isolates of *E*. *coli* and *Klebsiella* from our institution, only 79% and 65%, respectively, were susceptible to cephalosporins. Thus, the susceptibility rates among outpatient isolates for third generation cephalosporins in the current report are actually lower than those of ICU organisms in the US or Europe. The levels of antimicrobial resistance among Gram negative organisms, particularly community onset *K*. *pneumoniae* and *E*. *coli* are striking and emphasizes a growing appreciation of the urgency of addressing antimicrobial resistance [25] on a global basis. The level of resistance of *K*. *pneumoniae* to carbapenems and aminoglycosides is particularly alarming, since the polymyxins and sometimes tigecycline are currently the only effective alternatives.

In a systematic review of antimicrobial drug resistance among clinically relevant bacterial isolates in sub-Saharan Africa for community-acquired infections, the median susceptibility to third-generation cephalosporins ranged between 53.5% and 100% in West Africa, between 84.6% and 94.0% in central South Africa and between 78% and 100% in East Africa [26].

However, some studies from the region have found evidence for high levels of drug resistance among Gram negative rods. In a study of surgical site infections in Tanzania, 61.4% of the Gram negative rods isolated were multi drug resistant [27]. Another study in Western Kenya found a majority of the S. Typhi and S. Typhimurium isolates were resistant to at least 4 of the antibiotics used for treatment, however all were susceptible to ciprofloxacin [28]. A combination of factors may be contributing to the high levels of resistance demonstrated in the Gram negative organisms in the region. The lack of antibiotic stewardship in health facilities, misuse of antibiotics by the public and in veterinary practice, weak surveillance of antibiotic resistance and rudimentary infection control practices play a big role. Although prescriptions are legally required for antibiotics, the requirement is not enforced, so antibiotic use is common even among patients who don’t see physicians or other health care providers [29]. Previous studies within this hospital that determined the genotypes associated with MDR gram negatives found the majority of ESBL producing organisms had *bla*_CTX-M 15_. NDM1 was also isolated from seven Klebsiella isolates that were collected as early as 2007 [30].

The low frequency of Gram positive resistance provides a striking contrast to the high rate of Gram negative resistance. This is most notable for the low rate of MRSA, with 95% of *S*. *aureus* blood isolates and 94% of all *S*. *aureus* isolates in the current study being susceptible to oxacillin, compared with only 43.2% oxacillin susceptibility in a US survey of 1103 *S*. *aureus* isolates [31]. The other remarkable differences included the rarity of VRE in the current report as well as the lack of *S*. *pneumoniae* bloodstream isolates with high level resistance to beta-lactam antibiotics.

An additional contrast with many reports from elsewhere is the infrequent identification of *C*. *difficile* as a pathogen with a prevalence of 2.6% in requested tests. A systematic review found that the specificity of the test used varied from 94 to 99% in 9 reports, with an average of 97% [4]. Thus, the prevalence rate is low enough that these could all be false positive results. In addition, no cases of *C*. *difficile* colitis severe enough to proceed to colectomy have been identified (unpublished data). Thus, it remains to be determined whether *C*. *difficile* disease is found locally.

## Conclusion

We found similarities as well as notable differences in the patterns of microbial agents and antimicrobial resistance between our data and what has been reported from Europe and North America. Gram negative organisms were relatively more common as blood culture isolates. In addition, the resistance rates of both community onset and hospital onset infections were higher. Conversely, Gram positive organisms were less resistant, particularly exemplified by the low MRSA rates. The determinants for these differences are not obvious and a better understanding of the reasons may contribute to ongoing efforts to combat antimicrobial resistance globally.
